# Supplementary material for: Materialists perceive their high socioeconomic status as justice: Associations with increased political participation
Source: PLoS One. 2025 Jun 2;20(5):e0324680. doi: 10.1371/journal.pone.0324680 (PMC12129219; doi:10.1371/journal.pone.0324680)
Supplement: S1 File — Development of the Political Participation Behavior Scale. (DOCX) [file pone.0324680.s001.docx]

# Materialists Perceive Their High Socioeconomic Status as Justice: Leading to Increased Political Participation

**Supporting Information 1**

**Development of the Political Participation Behavior Scale**

This study aimed to develop and validate a self-reported scale to measure the political participation behavior of Chinese youth. Through conceptualization, literature analysis, and interviews, an initial item pool was generated. Subsequently, two rounds of sampling were conducted: the first for exploratory factor analysis and item analysis, and the second for confirmatory factor analysis and internal consistency testing. This process resulted in the development of a standardized Political Participation Behavior Scale.

## Materials and Methods

### Participants

This study conducted a total of two rounds of testing. Sample 1 consisting of 374 participants, was used for exploratory factor analysis and item analysis. The average age of the participants was *M* =20.95, *SD* =2.66, comprising 216 males (57.8%) and 152 females (40.6%), and 6 individuals who did not report their sex. Sample 2 with 102 participants, was employed for confirmatory factor analysis and assess internal consistency reliability testing. The average age of the participants was *M* =22.69, *SD* =1.05, comprising 55 males (53.9%) and 47 females (46.1%).

### Questionnaire Development Process

First, **Conceptualizing**. A literature review of existing political participation scales informed the operational definition of political participation. Political participation encompasses individuals' actions aimed at influencing governmental policies and decisions, including both offline activities (such as joining political parties, voting in local elections, and discussing social issues with others) and online forms (such as reporting social issues to the media, signing online petitions, and sharing or creating political content on digital platforms) [1-3].

Second, **Draft Initial Questions**. Subsequently, specific political behaviors related to youth were identified through interviews with college students, teachers, and student administrators, as well as a review of relevant literature. Based on the collected behaviors, a 13-item pool was generated, covering actions such as ‘visiting the official website of the government, relevant departmental websites, or government-affiliated new media’ ‘sending comments to the school through the Principal's mailbox’ ‘joining a political club at school’. Participants were asked to rate the frequency of each of the 10 items on a 5-point scale (1 = never, 2 = rarely, 3 = sometimes, 4 = often, 5 = always).

Third, **Revise the Questionnaire**. Exploratory factor analysis was conducted to extract the dimensions of the questionnaire with Sample 1, with items that did not meet statistical criteria being removed. The remaining items were then analyzed for item-total correlations, and those not meeting the criteria were discarded.

Fourth, **Validity and Reliability Testing.** The questionnaire was tested for construct validity using confirmatory factor analysis with Sample 2. The internal consistency reliability of overall scale.

### Statistical Methods

Exploratory factor analysis, item analysis, and internal consistency testing were conducted using IBM SPSS Statistics 22, while confirmatory factor analysis was conducted using Amos 24.0.

## Results and Discussion

### Exploratory Factor Analysis

Exploratory Factor Analysis (EFA) was conducted on 13 items using principal axis factoring with direct oblimin rotation. The results indicated that two factors had eigenvalues greater than 1. One item had a factor loading above 0.4 on Factor 2, while two other items had loadings exceeding 0.4 on both factors [4]. Consequently, these three items were removed.

A second exploratory factor analysis was then performed on the remaining 10 items. The results showed a Kaiser-Meyer-Olkin (KMO) value of 0.90, and Bartlett’s test of sphericity yielded *χ^2^*=2,079.62 (*p*<0.001), confirming the suitability of EFA. The analysis identified a single factor with an eigenvalue greater than 1, accounting for 55.60% of the variance, with factor loadings ranging from 0.68 to 0.82. Factor loadings for individual items are presented in S1 Table 1.

**S1 Table 1.** Factor Loadings for the Political Participation Behavior Scale

| Items | Factor |
| --- | --- |
|  | 1 |
| 1. Sending comments to the school through the Principal's mailbox | 0.70 |
| 2. Share opinions on school decisions through the school forum. | 0.78 |
| 3. Discuss concerns or advocate for benefits with the counselor or head teacher. | 0.69 |
| 4. Enjoy expressing opinions in politically relevant classes. | 0.68 |
| 5. Join a political club at school. | 0.75 |
| 1. Visiting the official website of the government, relevant departmental websites, or government-affiliated new media. | 0.70 |
| 7. Engage in online discussions about social issues or policies. | 0.76 |
| 8. Take part in online voting related to public events. | 0.79 |
| 9. Report complaints or allegations through online platforms. | 0.82 |
| 10. Contribute to policy formulation or revision by submitting opinions via official websites (e.g., providing feedback on amendments to the "Marriage Law" through the State Council's mini-program). | 0.77 |

***Item Analysis***

An item analysis based on total item correlation was conducted using data from Sample 1 to enhance the representativeness and reliability of the items in the final scale. The extreme group test method was employed and the independent samples t-tests revealed that each item could distinguish the response levels of different participants (all *p*<0.001), and Pearson correlation analysis showed that the correlations between each item score and the total score were all above 0.40 (*r* = 0.40~0.89, all *p*<0.001), suggesting high homogeneity between the items and the total scale [4]. In summary, it can be concluded that the discriminative power of all items was satisfactory, and thus all items were retained.

**S1 Table 2.** Item-Total Correlations and Item Discrimination

| Items | Item-Total Correlations | Item Discrimination |
| --- | --- | --- |
| Q1 | 0.70^**^ | -13.55^***^ |
| Q2 | 0.78^**^ | -17.38^***^ |
| Q3 | 0.70^**^ | -14.57^***^ |
| Q4 | 0.69^**^ | -14.97^***^ |
| Q5 | 0.75^**^ | -17.47^***^ |
| Q6 | 0.70^**^ | -17.19^***^ |
| Q7 | 0.76^**^ | -20.10^***^ |
| Q8 | 0.79^**^ | -21.80^***^ |
| Q9 | 0.82^**^ | -21.59^***^ |
| Q10 | 0.77^**^ | -19.44^***^ |

^**^*p*＜0.01, ^***^*p*＜0.001.

***Confirmatory Factor Analysis***

To test the structural validity, confirmatory factor analysis (CFA) was conducted using Sample 2. The results indicated an acceptable to good model fit [5]: *χ^2^/df*=1.80, *CFI*=0.96, *TLI*=0.93, and *RMSEA*=0.09.


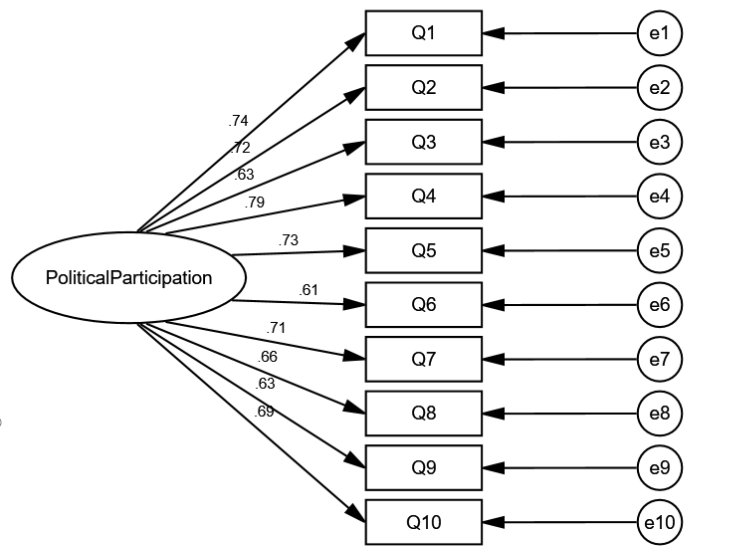


**S1 Fig. 1.** Structure Diagram of the Political Participation Behavior Scale.

***Internal Consistency Reliability***

Internal consistency for the scale was assessed using *Cronbach's α* coefficients based on data from Sample 2. The results indicated *Cronbach's α* values of 0.93 for the scale. A Cronbach's α ≥ 0.9 indicates excellent internal consistency [6], confirming the high internal consistency of the questionnaire.

## References

1. Wang Y, Wang H, Xie C, Wang XY. A college students’ online political participation: An analysis and discussion of their cognition and behavior: An empirical study of 10 universities in Zhejiang Province. Zhejiang Social Sciences. 2011;(5):132–139+105+160. doi:10.14167/j.zjss.2013.05.022.
2. Zhang HB, Wang MS. Comparison between Political Participation for University Students in Reality and on Internet in Contemporary China: an empirical study on 336 university students in Nanjing. Nanjing Journal of Social Sciences. 2011;(9):75–82. doi:10.15937/j.cnki.issn1001-8263.2011.09.024.
3. Lu JY. The Impact of Social Media on Young Adults’ Political Participation, and Moderation Effects of Internet Regulation: A Survey on Students from Nine Universities in China. Journalism & Communication. 2018;40(8):98–121. doi:10.13495/j.cnki.cjjc.2018.08.006.
4. Wu ML. Practical Guide to Questionnaire Statistical Analysis: SPSS Operations and Applications. Chongqing: Chongqing University Press. 2010.
5. Hu LT, Bentler PM. Evaluating model fit. In: Hoyle RH, editors. Structural equation modeling: Concepts, issues, and applications. Southern California: Sage Publications, Inc; 1995. pp. 76-99.
6. Nunnally JC. Psychometric Theory (2nd ed), New York: McGraw-Hill.1978.
