# Supplementary material for: Materialists perceive their high socioeconomic status as justice: Associations with increased political participation
Source: PLoS One. 2025 Jun 2;20(5):e0324680. doi: 10.1371/journal.pone.0324680 (PMC12129219; doi:10.1371/journal.pone.0324680)
Supplement: Attachment — Submitted filename: Response to Reviewers.docx [file pone.0324680.s005.docx]

# Response Letter to Reviewers

Dear reviewers,

We sincerely appreciate your valuable suggestions. In response to these comments, we have primarily made the following revisions to the manuscript: (1) A substantial number of references have been added to the Introduction and Discussion sections to strengthen the arguments. (2) We have further elaborated on the participant selection procedure and provided more information about the participants' demographic characteristics. (3) We have added details regarding the research methodology, including the questionnaire development process (see details in Supplementary Material), provided references for the criteria of all indicators and conducted a Structural Equation Model (SEM) analysis to test the moderated mediation model. (4) We have discussed the potential impact of Chinese culture on the research findings and generalizability of the results, as well as the practical applications. Additionally, we have carefully revised the manuscript to improve its clarity and facilitate better comprehension for readers. Our point-by-point responses are provided below.

If you have any further concerns, please do not hesitate to raise them.

Response to Reviewer #1

I am grateful for the opportunity to review this manuscript. It presents an interesting research topic. The authors have employed a quantitative research method to examine the relationships among the variables, which is refreshing.

**Response:** Thank you very much for your positive comments and detailed suggestions, which have significantly contributed to improving the quality of the manuscript. Our point-by-point responses are as follows.

Q1: The correlations presented in the abstract should reflect the correlations among all variables in the study, including the analysis of materialism value.

**Response:** Thank you for your suggestion. We have revised the abstract to include the correlations of all variables, including materialism value. The updated sentence reads:

*"Materialism value shows a significant negative correlation with SSS, perceived social justice, and political participation."*

(*See Abstract*)

Q2: The introduction and literature review would benefit from more literature evidence. Generally, statements that are not the authors' own opinions require citations. The lack of citations is a significant issue in this manuscript, a large lack of literature citations can lead reviewers to question the necessity and rigor of the study.

**Response:** Thank you for pointing out this issue. We have added 30 new references to support the statements, ensuring that each claim is appropriately cited. Since they are scattered throughout the manuscript, please refer to the revised citations in the manuscript. All newly added references have been highlighted.

(*See Introduction, Discussion and References*)

Q3: There are many instances in the introduction and literature review where it states "numerous studies indicate...". There should be at least three references to support this claim.

**Response:** Thank you for your suggestion. We have revised all similar instances throughout the manuscript and addressed them by adding multiple references to support such claims. For example:

*"* *Numerous studies have shown that materialism value significantly impacts individuals' perceived social justice [33-35]."*

*(See Introduction and Discussion)*

Q4: The proposed moderated mediation model (Fig. 1) should clarify whether the moderating variable has a positive or negative moderating effect.

**Response:** Thank you very much for your suggestion. We have revised Figure 1 (the proposed model) to clarify the directionality of the moderating effect. The figure now includes a "+" sign to indicate that materialism strengthens the positive relationship between SSS and perceived social justice.

*(See Fig.1)*

Q5: Please elaborate on the sample selection process or reason in the research methods, as this will shed light on sample representativeness.

**Response:** Thank you for your suggestion. We have expanded the explanation of the sample selection process in the Methods section. The revised text now reads:

" *The participants were recruited through two methods: first, data were collected via cluster sampling from courses, with students receiving course points as a reward for completing the survey; second, a snowball sampling technique [37] was employed, where students who had completed the survey invited their peers from different universities to participate. This approach ensured that the sample included participants from both top-tier and regular universities, as well as from a diverse range of regions and institutions, thereby enhancing the representativeness of the sample. The homogeneity of university students allowed for more effectively control of confounding variables, which strengthened the examination of relationships among various variables in the model being tested [38]."*

(*See Participants*)

Q6: What is the basis for the minimum sample size, and how to control the bias in advance (please note that Harman’s single-factor test is a post-hoc bias test)? For further learning on bias control, you may refer to the following literature:

Podsakoff, P. M., MacKenzie, S. B., Lee, J. Y., & Podsakoff, N. P. (2003). Common method biases in behavioral research: a critical review of the literature and recommended remedies. Journal of applied psychology, 88(5), 879.

**Response:** Thank you very much for raising this question.

Regarding the minimum sample size, when using PROCESS, a sample size of 5-10 times the number of items is typically sufficient (Wolf et al., 2013). For this study, with 32 items, a sample of 320 participants would be sufficient. However, the larger the sample size, the more robust the results. Therefore, the initial design of this study aimed for a sample size of 1000 participants, and during implementation, this number was exceeded, with the final valid sample size being 1306.

Regarding bias control, we greatly appreciate the reference you provided, which is very helpful for both this study and our future research design. As a self-reported questionnaire study, this study controlled for potential bias through procedures such as eliminating item ambiguity, anonymous testing, and conducting Harman's single-factor test. However, method bias may still affect the results. We have discussed this issue in the Limitations and Future Research section.

*“Forth, this study is a self-reported questionnaire study, which may be subject to common method bias [53]. Although the study controlled for this potential issue through procedures such as eliminating item ambiguity, anonymous testing, and conducting Harman's single-factor test, method bias may still affect the results. Future research could employ more diverse measurement methods, such as longitudinal designs, behavioral indicators, and experimental designs, to examine the relationships between variables and eliminate the impact of method bias on the results.”*

(*See Limitations and Future Research*)

**Reference for the response:** Wolf EJ, Harrington KM, Clark SL, Miller MW. Sample Size Requirements for Structural Equation Models: An Evaluation of Power, Bias, and Solution Propriety. Educational and Psychological Measurement. 2013;73(6):913-34. doi: 10.1177/0013164413495237.

Q7: What platform was used for online questionnaire collection? Please provide a valid link to that platform.

**Response:** Thank you for pointing out the issue. We have clarified the platform details in the Methods section:

"*Data were collected via Tencent Questionnaire (https://wj.qq.com), a widely used platform in China.*"

(*See Participants*)

Q8: More demographic background information about the participants should be provided, such as their grade and family location, as these background variables may affect the stability of the research results.

**Response:** Thank you for your suggestion. We have added additional demographic background information, including grade and family location distributions. The revised information now specifies:

*"* *The participants were 721 males (55.6 %), 577 females (44.2 %), and 2 individuals did not report their sex. The mean age of the participants was 20.46 years (SD= 1.21). The grade distribution included 743 freshmen and sophomores (56.9%), 454 juniors and seniors (34.8%), and 107 graduate students (8.2%), with two participants not reporting their grade. Participants were from 16 of China's 34 provinces, autonomous regions, and municipalities, with 881 (67.5%) from urban areas, 422 (32.3%) from rural areas, and 3 not reporting their home location."*

(*See Participants*)

Q9: I noticed that the other three measurement tools used by the authors are Likert scales. Is the SSS scale also a Likert scale? If not, how are the scores converted of SSS scale? Additionally, please provide reliability and validity analysis for the SSS scale.

**Response:** Thank you for raising this question. The MacArthur Scale uses a 10-point ladder (not Likert). Scores are treated as continuous. Its validity is supported by Adler et al. (2000), cited in the Methods section. We have also added the following explanation to clarify the nature of the SSS scale and its scoring method:

*"* *Scores are treated as continuous variables, and the scale's validity and reliability have been well-documented in previous research [12]."*

(*See SSS*)

Q10: As far as I know, developing a self-constructed questionnaire or scale requires substantial preliminary work, such as: how to generate an item pool? Is item analysis and exploratory factor analysis conducted? To ensure the reliability and validity of the tool. Although this study uses CFA, it lacks the necessary steps for a self-constructed questionnaire, which raises doubts about the measurement tool.

**Response:** Thank you very much for raising this question. The questionnaire was developed following a rigorous process, which included item pool generation, item analysis, exploratory factor analysis (EFA), and confirmatory factor analysis (CFA). These steps are critical for ensuring the reliability and validity of a newly developed scale.

To maintain conciseness in the manuscript, we opted to summarize these results in the main text while providing the full methodological details and outputs in the *Supporting Information 1 (Development of the Political Participation Behavior Scale)*. All psychometric indices (e.g., factor loadings, reliability) met conventional thresholds for validity and reliability.

We are happy to provide further clarification or additional data if needed.

(*See Political participation*)

Q11: Please provide the criteria or reference ranges for all indicators in the research methods and results, such as Cronbach's α, model fit indices, and the critical threshold of 40%.

**Response:** Thank you for pointing out the issue. We have provided the criteria or references for all indicators in the manuscript. For examples:

*"* *In this study, Cronbach's α was 0.81, which is greater than 0.7, indicating that the internal consistency reliability is acceptable [39]."*

*"* *According to the criteria, χ2/df < 3, CFI and TLI ≥ 0.90, and RMSEA between 0.08 and 0.10 indicate a good fit[41]. "*

(*See Measures*)

Q12: Please detail the data analysis strategy used in this study.

**Response:** Thank you for your suggestion. We have added a subsection titled "Data Analysis " to the Methods section:

" *Preliminary analyses, including Harman's single-factor test, descriptive statistics, and Pearson correlation analysis, were conducted using IBM SPSS Statistics 22. Subsequently, the moderated mediation model was tested using the PROCESS macro for SPSS. Bootstrapping with 95% confidence intervals was employed to assess the statistical significance of the moderated mediation model, based on 5000 random samples [42]. Additionally, we conducted a Structural Equation Model (SEM) analysis using AMOS 24.0 to validate the validity of the moderated mediation model.*"

(*See Methods*)

Q13: The descriptive statistics and correlation analysis of the research results should include the moderating variable, materialism value.

**Response:** Thank you very much for pointing out the issue. We have updated Table 1 to include means, standard deviations, and correlations for materialism value.

(*See Table 1*)

Q14: The discussion section would benefit from more literature support, and incorporating discussions on the Chinese cultural context could further explain the validity of the research results.

**Response:** Thank you very much for your suggestion. We have expanded the discussion to incorporate China's sociocultural context. Please refer to:

*"Given the inconsistent findings on the relationship between socioeconomic status (SES) and political participation in previous research, this study’s results may hold unique significance within China’s sociocultural and economic context. These factors shape how individuals perceive their socioeconomic status and, in turn, influence their actions. In China’s collectivist culture, high-SSS individuals often align their personal success with national progress, viewing political participation as a civic duty to maintain social harmony [45]. This contrasts with individualistic Western societies, where high-SSS groups may prioritize self-interest over collective action [46]. Studies show that Confucian values, such as loyalty to the state and an emphasis on social order, strengthen the link between SSS and political engagement [47]. For example, urban Chinese with higher SES are more likely to endorse government policies, perceiving their participation as a contribution to national stability [48]. Additionally, China’s high levels of political trust, particularly among educated and urban populations, reinforce the SSS-political participation link. Unlike in democracies where distrust in government may drive low-SSS groups to protest [15], Chinese high-SSS individuals tend to perceive the government as a legitimate arbiter of social mobility. Government-led campaigns, such as poverty alleviation programs and anti-corruption drives, are framed as evidence of systemic fairness, encouraging participation [23]. For instance, a 2020 survey found that 68% of high-SSS Chinese viewed local governance as “responsive,” compared to 42% in a comparable U.S. cohort [9]. Moreover, the Chinese government strategically channels political participation through state-sanctioned platforms (e.g., online petitions, youth leagues), which are more accessible to high-SSS individuals with resources and education [32]. This contrasts with Western settings, where political engagement often requires independent mobilization [21]. These factors may collectively shape the positive relationship between SSS, perceived social justice and political participation in China. Therefore, the generalization of this study’s findings should take multiple factors into account."*

(*See Discussion*)

Q15: Please clarify the practical value of this research.

**Response:** Thank you for your suggestion. We have added a subsection titled "Practical Implications" to the Discussion section, which emphasizes policy and educational interventions (e.g., promoting intrinsic values, equitable resource distribution). This addition highlights the real-world applications of the study's findings. Please see the updated section below:

*“The findings of this study have significant implications for policymakers and educators. First, the mediating role of perceived social justice suggests that interventions aimed at enhancing fairness in resource distribution—such as transparent governance and equitable access to education—could mitigate political apathy among low-SSS groups, thereby promoting greater engagement. For example, grassroots campaigns emphasizing social equity (e.g., anti-corruption initiatives) may foster trust in institutions and encourage participation [23]. Second, the moderating effect of materialism highlights a dual challenge in China’s rapidly modernizing society, where economic growth raises material aspirations, yet excessive materialism may undermine collective values essential for civic engagement [32]. Educational programs promoting intrinsic values (e.g., community service, ethical leadership) could counterbalance materialistic tendencies, particularly among youth. For example, integrating Confucian principles of social harmony into curricula might strengthen the link between SSS and prosocial political behaviors [52]. Third, the study’s focus on university students—a demographic central to China’s future governance—underscores the need for campus initiatives that bridge socioeconomic divides. Such initiatives could include policies like financial aid for low-SSS students or platforms for inclusive political discourse (e.g., student councils), both of which would help reduce perceived inequities and empower marginalized groups to engage more actively [47].*”

(*See Discussion*)

In summary, this manuscript requires more effort in literature citations to meet the standards of academic writing.

**Response:** Thank you once again for your valuable comments and suggestions, which have been extremely helpful in revising our manuscript. If you have any further suggestions for improvement, we welcome them and would be happy to address them.

Response to Reviewer #2

The study provides valuable insights into the interplay of socioeconomic perceptions, justice beliefs, and material values in shaping political participation.

General Feedback:

The paper presents a well-structured investigation into the relationship between subjective socioeconomic status (SSS) and political participation. The findings contribute meaningfully to political psychology and behavioral economics by integrating psychological mechanisms such as perceived social justice and materialism. The use of established scales and robust statistical techniques enhances the credibility of the study. However, certain areas require further clarification and elaboration to strengthen the manuscript.

**Response:** Thank you very much for your positive comments and constructive suggestions, which have been very helpful in revising our manuscript. We have carefully revised the manuscript based on your feedback. Our point-by-point responses are as follows.

**Major Points:**

The authors acknowledge contradictory findings in the literature regarding SSS and political participation. While this study finds a positive relationship, some studies suggest a negative or null effect. Could the authors provide a more detailed discussion of why their findings differ from others? Are contextual factors (e.g., China's political environment, university culture) potentially influencing this relationship?

**Response:** Thank you very much for pointing out this issue. We have added an analysis of these contradictory findings when presenting our hypothesis:

*“The results indicating a negative relationship between SSS and political participation primarily point to forms of resistance or disruptive political engagement [17, 24, 25]. In contrast, studies suggesting a positive relationship tend to focus on more moderate, non-confrontational forms of political participation and offer a more thorough analysis of the psychological processes underlying the relationship between SSS and political participation. In the current stable political environment in China, political participation among youth is generally more moderate [26]. Based on these considerations, we present Hypothesis 1: SSS is positively correlated with political participation.”*

We have also incorporated an analysis of the results in the discussion, considering the Chinese cultural context.

*"* *Given the inconsistent findings on the relationship between socioeconomic status (SES) and political participation in previous research, this study’s results may hold unique significance within China’s sociocultural and economic context. These factors shape how individuals perceive their socioeconomic status and, in turn, influence their actions. In China’s collectivist culture, high-SSS individuals often align their personal success with national progress, viewing political participation as a civic duty to maintain social harmony [45]. This contrasts with individualistic Western societies, where high-SSS groups may prioritize self-interest over collective action [46]. Studies show that Confucian values, such as loyalty to the state and an emphasis on social order, strengthen the link between SSS and political engagement [47]. For example, urban Chinese with higher SES are more likely to endorse government policies, perceiving their participation as a contribution to national stability [48]. Additionally, China’s high levels of political trust, particularly among educated and urban populations, reinforce the SSS-political participation link. Unlike in democracies where distrust in government may drive low-SSS groups to protest [15], Chinese high-SSS individuals tend to perceive the government as a legitimate arbiter of social mobility. Government-led campaigns, such as poverty alleviation programs and anti-corruption drives, are framed as evidence of systemic fairness, encouraging participation [23]. For instance, a 2020 survey found that 68% of high-SSS Chinese viewed local governance as “responsive,” compared to 42% in a comparable U.S. cohort [9]. Moreover, the Chinese government strategically channels political participation through state-sanctioned platforms (e.g., online petitions, youth leagues), which are more accessible to high-SSS individuals with resources and education [32]. This contrasts with Western settings, where political engagement often requires independent mobilization [21]. These factors may collectively shape the positive relationship between SSS, perceived social justice and political participation in China. Therefore, the generalization of this study’s findings should take multiple factors into account."*

(*See Introduction,* *Limitations and future research*)

The study suggests that individuals with higher SSS perceive greater social justice, which in turn fosters political participation. However, previous research indicates that individuals with higher SSS might disengage from political participation due to a sense of complacency. Can the authors explore alternative explanations for the mediation effect? Could there be a nonlinear or curvilinear relationship that was not tested? However, it is possible that relationships are more complex. The authors did not test for any non-linear relationships and assume linear relationships between variables. For example, the relationship between SSS and political participation may not be consistently positive. It is possible that at very high levels of SSS, individuals may become complacent or disengaged from political activities. Similarly, there might be a threshold effect where only after a certain level of perceived social justice has been reached do individuals become more politically active. This might result in a curvilinear relationship, which the study did not explore.

**Response:** The issue you raised is highly important. This study assumes linear relationships between variables and focuses solely on linear effects. The primary reason for this approach is that our research aims to examine the mediating role of perceived social justice and the moderating effect of materialism. If the relationship between the independent and dependent variables were nonlinear, the interaction effect between the independent variable and the moderator would become exceedingly complex and analytically intractable.

Nevertheless, we acknowledge that the relationship between SSS and political participation may be more complex than a simple linear association. This presents a valuable direction for future research, which we have now explicitly addressed in the "Limitations and Future Research" section:

*"On the other hand, nonlinear relationships may exist. For example, at extreme SSS levels, individuals might disengage due to overconfidence in systemic fairness or resource saturation. Future studies should test quadratic or moderated nonlinear models to capture these nuances."*

(*See* *Limitations and future research*)

The findings indicate that materialism strengthens the relationship between SSS and perceived social justice. However, materialism is a multidimensional construct with facets such as success, centrality, and happiness. Although the study does not explore whether all three dimensions of materialism have different moderating effects, it is plausible that they do. Future research could explore these differences to develop a more comprehensive understanding of how materialism influences the relationship between SSS, perceived social justice, and political participation. Do all dimensions of materialism exert the same moderating effect? If not, how do they differ?

**Response:** Thank you very much for your suggestion. We conducted separate analyses for each dimension of materialism and found that the moderating effects of centrality and happiness were significant, while the moderating effect of success was not. We have reported these findings in both the results and discussion sections.

*“The moderating roles of three materialism value dimensions (success, centrality, and happiness) on the relationship between SSS and perceived social justice were also tested. We used PROCESS Model 7, controlling for gender and age in all analyses. For material success, the interaction with SSS was not significant (β = 0.04, p = 0.081), indicating no moderation effect.* *In contrast, material centrality significantly moderated the relationship: its interaction with SSS was significant (β = 0.06, p < 0.05), with a stronger effect in high-centrality individuals (β = 0.14, p < 0.001) than low-centrality individuals (β = 0.02, p = 0.626). Similarly, material happiness significantly moderated the relationship:* *its interaction with SSS was significant (β = 0.05, p < 0.05). High-happiness individuals showed a significant SSS–justice link (β = 0.11, p < 0.01), while low-happiness individuals did not (β = -0.004, p = 0.93). Bootstrap analyses confirmed moderated mediation for both centrality and* *happiness, with indirect effects significant only in high-materialism groups (indirect effect _centrality_ = 0.03, 95% CI [0.01, 0.05]; indirect effect _happiness_ = 0.03, 95% CI [0.01, 0.05]). Thus, centrality and happiness—but not success—played a moderating role.”*

*“Notably, two of the three dimensions of materialism—centrality and happiness—play distinct roles in moderating the SSS–justice link. Material centrality, which prioritizes possessions as the core purpose of life, amplifies the belief that SSS reflects personal merit [36]. Individuals with high material centrality are more likely to interpret their socioeconomic status as evidence of a fair system, a pattern particularly observed among urban Chinese youth [32]. Similarly, material happiness, which associates well-being with access to material resources, reinforces perceptions of fairness among high-SSS individuals [35]. In contrast, material success, which reflects the belief that personal achievement is closely tied to the accumulation of wealth and possessions, does not significantly moderate the SSS–justice link. This may be because material success emphasizes individual accomplishment rather than social comparison [51]. As a result, individuals with strong material success values may less likely to attribute low SSS to social injustice and do not exhibit an enhanced perception of justice at high SSS levels, which may explain the absence of a moderating effect.”*

(*See Results, Discussion*)

The study relies on PROCESS models which are well-established but have limitations. Could the authors consider alternative methods such as Structural Equation Modeling (SEM) or Bayesian Mediation Analysis? By using alternative methods like SEM or Bayesian analysis, the authors could gain a more nuanced and comprehensive understanding of the complex relationships among SSS, perceived social justice, materialism and political participation. These methods would provide a more robust assessment of the model's validity and offer the possibility of exploring more complex relationships. A comparison of methods could add robustness to the findings.

**Response:** Thank you for your thoughtful suggestion regarding methodological rigor. We agree that employing complementary analytical approaches can strengthen the validity of the findings. In addition to the PROCESS models, we conducted a Structural Equation Model (SEM) analysis using Amos 24.0 to test the moderated mediation model. The SEM results demonstrated excellent model fit indices (χ²/df = 1.72, RMSEA = 0.02, CFI = 0.99, TLI = 0.97). We have supplemented the manuscript with this additional reporting.

*“To strengthen the validity of the findings, a structural equation model (SEM) was constructed to test the moderated mediation model. The results demonstrated a good fit to the data (χ2/df=1. 72, RMSEA=0. 02, CFI=0. 99, TLI=0. 97)[41].”*

(*See Results*)

Additional Points for Consideration:

• Would a more heterogeneous sample yield different findings?

**Response:** You raised a very valuable point. A more heterogeneous sample could indeed lead to different results, and it aligns with our future research direction. We have addressed this point in the limitations and future research section.

*"Finally, our homogeneous sample (Chinese university students) limits generalizability. In more diverse populations (e.g., in other countries or cultures, or among older adult groups), SSS might correlate differently with political participation due to varying access to resources or exposure to state narratives [54]. Cross-cultural replications are critical: in individualistic societies, materialism might weaken the SSS–justice link by prioritizing self-interest over collective fairness [32]. Comparative studies could disentangle cultural versus structural influences. Future research could include participants from different socioeconomic and cultural backgrounds."*

(*See Limitations and future research*)

• Given China's unique political and economic landscape, to what extent do the findings generalize to other cultural settings?

**Response:** Thank you very much for your valuable feedback. In the discussion section, we have examined the influence of the Chinese cultural context and the potential for generalization. Please refer to our responses to the questions on contextual factors and heterogeneous samples.

(*See Discussion, Limitations and future research*)

Overall, this study offers valuable contributions to the understanding of political participation among Chinese college students. The use of a moderated mediation model is commendable, but further clarifications, additional robustness checks, and theoretical discussions would enhance the manuscript. I encourage the authors to address these points in their revision.

**Response:** Thank you once again for your valuable suggestions. We hope that these revisions address your concerns. If you have any further comments or suggestions, please feel free to share them, we welcome them and would be happy to address them.

Sincerely,

The Authors
