# Supplementary material for: Materialists perceive their high socioeconomic status as justice: Associations with increased political participation
Source: PLoS One. 2025 Jun 2;20(5):e0324680. doi: 10.1371/journal.pone.0324680 (PMC12129219; doi:10.1371/journal.pone.0324680)
Supplement: Attachment — Submitted filename: Response to Reviewers-2nd.docx [file pone.0324680.s006.docx]

# Response Letter to Reviewers

Response to Reviewer #1

I would like to express my appreciation for the authors’ efforts in revising the manuscript. The quality of the revised version has been significantly improved, and the authors have adequately addressed my concerns. It is evident from the revised manuscript and the response letter that the research team has dedicated considerable effort to this work. However, given that your study is cross-sectional in design, I recommend revising the title to remove the term “lead to,” which. Once this modification is made, I am prepared to recommend the manuscript for publication.

**Response:** Thank you very much for your positive evaluation of our manuscript. Your comments have been extremely helpful in improving our paper.

We agree with your concern that the phrase “lead to” may imply a causal relationship. After discussion, we have revised the title to “*Materialists Perceive Their High Socioeconomic Status as Justice:* ***Associations with*** *Increased Political Participation*” to reflect a correlational rather than a causal relationship.

We sincerely hope that our revisions address your concerns. Should you have any further questions, please do not hesitate to contact us.

Sincerely,

The Authors
